# What Medicine And Medical Journal Editing Mean To Me

**DOI:** 10.4103/0973-1229.27606

**Published:** 2006

**Authors:** Shaukat Ali Jawaid

**Affiliations:** * *Chief Editor, Pulse International, Also Managing Editor, Pakistan Journal of Medical Sciences, Panorama Centre, Building No.2, Room No.522, Raja Ghazzanfar Ali Road, Sadder, Karachi: Pakistan. Tel: 92-021- 5688791, 92-021- 5689285. Fax: 92-021-5689860. E-Mail: shaukat@pulsepakistan.com.*

**Keywords:** *Medicine*, *Medical Journalism*, *Medical Editor*

## Abstract

Medicine and medical journalism are both noble professions. Those who are infected with the materialistic virus and want to make quick money should not take up these as professional careers. Editing a good quality peer reviewed medical journal in a developing third world country is extremely frustrating. An editor has to work under considerable stress and strain, and face numerous pressures. However, it is a joy and pleasure to be a successful medical editor. The mere fact that one can help so many authors and influence decision makers in the medical profession, health officials, pharmaceutical trade and industry and all others connected with the health sector gives tremendous professional satisfaction, which is invaluable and keeps one motivated.

## Introduction

I have always believed that medicine as well as journalism are both noble professions and anyone who wishes to make quick money should never take up these fields as professional careers. In the practice of medicine, one gets the prayers and blessings of patients who are looked after; and seeing some one overcome disease to become a useful healthy member of society once again is, indeed, satisfying. These feelings of professional satisfaction - whether one successfully treats a patient, teaches and trains students and juniors, or gets involved in useful research - keeps one stimulated and motivated.

My entry in the field of medical journalism was a pleasant accident. I was keen to become a doctor but ended up becoming a medical editor. Even otherwise, for people like me who come from a lower middle-class background, planning a career is a luxury we can ill afford, as one is at the mercy of circumstances. However, sincerity to one's selected field and devotion pays; and the end result is a precious diamond well cut out from a rough edged stone. God Almighty has always been very kind to me and my wish to be a doctor was fulfilled to a great extent through all of my three children who became doctors, and are currently practicing in the fields of Medicine, Surgery and Psychiatry.

Medical journalism is a highly specialized field. Editing a quality peer reviewed medical journal in a developing Third World country is at times frustrating and a highly stressful job. It is just like giving birth to a baby, a very painful but equally pleasurable activity. Anyone going through this exercise once eventually relishes it so much that one keeps on doing it again and again.

Editors of peer reviewed medical journals face enormous problems (Jawaid, 2004). Since the subjects of medical writing and research methodology are not taught in our medical schools, healthcare professionals are not fully aware of it (Al-Ateeg, 2004; Jawaid, 2003). However, now the situation is changing rapidly. Some medical schools have included this subject in the curriculum, while postgraduates are also getting familiar with it, as they have to write dissertations or theses. Faculty members in medical institutions need some published papers for selection and further promotion; hence they too have to write at least a few papers. As it is done under compulsion, the quality of the papers may not be what is desired (Jawaid and Jafary, 1997). Very little good quality research is done in the medical institutions in developing Third World countries, since the researchers neither get any incentive nor rewards, something that needs to be looked into by all those concerned (Jawaid and Jafary, 1995).

The problems one faces while editing a medical journal are as outlined below.

## Problems Related To Authors

Most of the contributors do not read *Instructions to Authors*. They are not aware of Uniform Requirements for Submission of Manuscripts to Biomedical Journals as approved by International Committee of Medical Journal Editors (ICMJE, 2006; see their website at *http://www.icmje.org/*; also see especially for manuscript preparation: *http://www.icmje.org/ index.html#manuscript/*). Hence it is not uncommon to find that at times the total of numbers in tables, abstract and the manuscript differ, percentages calculated are wrong, legends of figures, illustrations are missing, or references are not cited in the manuscript. Some even do not know how to write a structured abstract. Presentation itself is extremely poor, hence not suitable to be sent for peer review. Poor English in some of the manuscripts, incorrect statistical analysis, failure to respond to queries in time or communicating change in address, incomplete references where even the editors and reviewers cannot do much, are some of the other problems which make the life of an editor miserable.

## Finding Good Quality Reviewers

One has to be a good writer before one can review a manuscript, and finding good quality reviewers is not easy. Co-ordination between authors and reviewers is an uphill task. Keeping track of manuscripts, sending reminders to reviewers, satisfying authors is equally difficult. At times, the quality of the review may not be satisfactory, or the reviewer may fail to give clear guidance as regards approval or rejection of the manuscript, necessitating it to be sent to another reviewer. One has to be constantly on the look out to update the reviewer's database. Editing a journal is a team-work. A journal is as good as its Editorial Board helped by the reviewers. Since most of the reviewers do this job honorary, editors cannot be strict with them to keep to the deadlines.

## Indexation Problems

Getting indexed in *Medline* is not easy for many journals. At present, only three out of about forty medical and dental journals published from Pakistan are included in *Medline*. However, over a dozen are covered by *EMBASE*, while quite a few are covered by *ExtraMED*, the *Index Medicus* of the WHO EMRO region. Some authors are always eager to get manuscripts published in journals covered by *Medline* as it ensures greater citations; hence attracting good quality manuscripts is a problem.

The situation in India appears to be much better with a large number of journals covered by *Medline.* My interaction with many Indian authors and researchers gives me an impression that they are imbibed with a nationalistic spirit; hence they feel proud to publish their research work in their own journals. We expect similar practices in Pakistan. Unless authors publish good quality manuscripts in their own journals, how is their quality going to improve?

## Failure to Disclose Source of Funding; Approval from Ethics Committee

During the last ten years, the situation has changed a lot. Now authors are required to disclose their source of funding, if any, and also get their manuscript approved by the Institutional Ethics Committee and Institutional Review Boards (IRBs). If no such body exists, approval from any regional or national body is considered a must. However, in many cases, investigators do not meet these requirements, creating lot of ethical problems for editors while reviewing and editing such manuscripts.

## Financial Viability

A vast majority of healthcare professionals have not developed a habit of reading, hence they never subscribe to a journal, and particularly so from their own countries. Libraries have no funds to subscribe to journals, either because institutions do not give it any priority, or no funds are allocated for this purpose. Advertising remains the major source of income but the pharmaceutical industry is more inclined to influence the doctors′ prescribing habits by looking after them in so many other ways, including all sort of unethical marketing practices. Hence advertising in journals is not on their priority list, either. At times, even if they do advertise, they try to influence the editorial policy despite the fact that advertisements placed by the pharmaceutical industry in medical publications all over the world is considered a legitimate support which is to the mutual advantage of industry as well as medical publishers. Hence an editor has to be extremely careful not to compromise on the credibility of the journal by succumbing to any of such pressures. I have had my share of such problems, but God Almighty gave me the courage and inner strength to face such situations.

### Case Study

In 1991, we received a case report from a junior faculty member of a postgraduate medical institution highlighting the adverse effects with overdose of Halofantrine. One of his patients had developed ventricular fibrillation and generalized convulsive seizures after administration of this anti-malarial at the recommended dosage. Electrocardiogram showed prolonged QT-interval with multifocal ventricular ectopic beats, which reverted spontaneously. He recommended that this drug be prescribed in a dose of 25mg/Kg body weight. When the company was approached to find out if they had any such information (because of the information they get during post marketing surveillance), we were told that this case report was earlier sent to an Indexed journal which had rejected it. Hence, we should not publish it. However, we went ahead with the peer review process and both the reviewers recommended its publication. While the journal was still under publication, one of the reviewers informed us that his own uncle, who was a cardiac patient, had died due to this antimalarial which was prescribed to him by their family physician. Had he not reviewed this case report, he wouldn't have known about it. He felt that this case report be published as soon as possible to save more lives. Its publication (Bukhari, 1991a) spoiled our relationship with the company, which opined that a local dose finding trial conducted in Pakistan by two senior most physicians had not noted any such adverse effects. Our publications were black listed by the said company and were not to receive any advertisements. Keeping up professional ethics, we offered them to print their viewpoint as well, to which they agreed. However, we also approached the author to get his viewpoint to the company's version, which was also incorporated, in the same issue, which further annoyed the company. (Yazdani, 1991; Bukhari, 1991b). Few months later, a report appeared in a publication of the Pakistan Medical Research Council from Peshawar reporting sudden death while on Halofantrine treatment (Akhtar and Imran, 1992). After two years, *The Lancet* published similar findings (Nosten *et al,* 1993) and the company was forced to write, *“Dear Doctor”* letters all over the world informing the healthcare professionals to be careful while prescribing this drug to heart patients as it could prolong the QT-interval. However, we had to suffer for all those three years, which was a tremendous financial loss. But it all feels worthwhile now, for our principled stand was vindicated.

## Duplicate Submission

Most standard peer reviewed journals insist that authors give a written undertaking regarding exclusive submission and confirm that the manuscript submitted is an unpublished material. Even after giving such a written undertaking, some authors do indulge in simultaneous submission to more than one journal. Editors have to trust the authors and they cannot act as policemen. Sometimes, such duplicate submissions are detected before the manuscript is published. But in other cases, it may escape the attention of reviewers as well as the editor.

When I had to take action of black listing such authors and also decided to convey the decision to their employers as well as concerned government agencies, departments, regulatory bodies like Pakistan Medical and Dental Council, it was a very unpleasant experience. In such situations, an editor should be prepared to face the wrath of such unethical authors. It could even pose a serious threat of physical assault, including security of life. Here again I had my share of such episodes (Jawaid and Jafary, 2005).

### Case Studies

1. Dr. A had submitted a manuscript for publication and also gave a written undertaking regarding exclusive submission to our journal. He was helped by the reviewers to re-write and revise the manuscript. However, he got it published in another journal without withdrawing it from our journal. We came to know about it while the journal was being printed. We had to delete that manuscript, re-number the pages before printing, all at a great financial loss. We wrote to the author who stated that he had never sent this manuscript to the other journal and he was not at all aware of its publication. He was obviously lying: how could the other journal get a manuscript unless the author provided it? We informed about this unethical practice to his employers, the institution head, Pakistan Medical and Dental Council as well as the Higher Education Commission. The author wrote us a letter stating that his professional career was at stake and if anything happened, he wouldn't spare us. We were prepared to face the consequences but luckily there were no further developments.
2. In yet another case, a faculty member submitted a manuscript and wanted to get it published quickly. He was worried since his retirement was due within a couple of months, his wife had already been promoted as Professor and he did not want to retire as Associate Professor. He even approached about half a dozen senior professors to ensure that his manuscript was accommodated early. As a special case, we got it reviewed and processed on a priority basis and published it after due peer review process. However, soon we were informed that two of the tables and a paragraph were copied from another article published by another author some years ago. Somehow we could not detect that fraud during the peer review process. We retracted that article and also informed about it to the author, his institution head, provincial health department and the Pakistan Medical and Dental Council. The author suffered a severe myocardial infarction and was admitted to the Coronary Care Unit. His wife made a threatening phone call saying that if anything went wrong with her husband, she would sue us. Luckily he survived and eventually retired as Associate Professor.

Nothing could be more frustrating for an editor than to find out that after an author has been helped to revise and improve his/her manuscript, the authors send it to some other journal just to get it published early. Or for them to ask that their manuscript be withdrawn at such a late stage that the paper has already been scheduled to be included in a forthcoming issue, or is already in the press.

## Rejection Of A Paper

As an editor, I am not happy, in fact, it is always painful to reject a paper that fails to meet the minimum required standard, or suffers from serious deficiencies. But I believe rejection of a manuscript is just like amputation, which can be life saving. It acts as a barrier to dissemination of wrong information. I believe an editor, who is not knowledgeable and is inexperienced, can be a very dangerous person; he can mislead and misguide many, and that can have serious repercussions.

## A Good Medical Editor

Hugh Clegg, Editor of the *British Medical Journal* (1947–1965) had stated:

*A medical editor has to be the keeper of the conscience of a profession and if he or she tries to live up to this ideal, he/she will always be getting into trouble* (Jawaid, 2004).*

It is generally felt that in order to have a never-ending quest for quality, a good editor should be surrounded by people who are more intelligent, have more expertise and are more skilled than he is. This is what all editors should aspire to achieve.

### The Most Frequently Asked Question

Every author wishes to see his name in print. Hence as soon as authors submit a manuscript, they start enquiring as to when it will be published. Many of them are not fully aware of the whole peer review process, which may take from few weeks to few months, depending on the working and database of various journals. My answer to such enquiries has always been:

You are trying to discuss the sex of the baby even before proper engagement.

Even after engagement, it take time for the marriage ceremony to take place, then the conception, which is followed by taking care of the fetus not only by the pregnant lady but the whole family, including her husband and the treating physician. All of them have to play their role for nine months to ensure positive outcome of the pregnancy. If they are not careful, many mishaps could occur i.e. abortion, intrauterine death of the baby, premature birth etc. All of which can be avoided with a caring attitude on the part of all concerned.

Similarly from writing and submission of a manuscript to its final publication, there are many people involved, i.e. editor, reviewers, publishers, and the authors themselves. The authors must respond to queries from the reviewers immediately, revise and resubmit the manuscript incorporating the comments and suggestions of the reviewers, clarify certain points if asked for by the editor, and return the manuscript promptly after proof reading. Any delay on the part of either reviewers or the authors in discharging their duties could result in unnecessary delay in final publication. The authors have to be patient and understanding of the editor's problems and difficulties. Similarly, the editors too should be accommodating and helpful.

### Pressures On An Editor

If the editor is not the owner of the journal as well, which is the case in most situations, he or she has to work under lot of pressures. More recently, editorial independence has become an important issue, which the editor must negotiate carefully before accepting the responsibility (Jawaid and Jafary, 1999). My observations have been that an editor is faced with pressure from the financial point of view, pressure from authors, reviewers and referees, readers, printers and technical editors, owners of the journal, advertisers, public institutions and government health authorities, not to forget the likelihood of physical assault and security of life (Jawaid, 2004). All this makes the job of an editor quite difficult.

Prof. Abdus Samad, a well-known interventional cardiologist of Pakistan and Editor of *Pakistan Journal of Cardiology,* defines the editor in this way:

The poor editor is just like a father who not only has to educate his daughter, arrange her jahez*, look for a son-in-law, but also find a suitable job for him, keep him in the house, provide him living and clothing, and also teach him how to eat and live a decent life (Jawaid, 2004).

Authors expect editors to do everything even if it means re-writing the manuscript to make it acceptable for publication, which many editors can ill afford to do.

## Pakistan Medical Journalists Association

I was instrumental and played a pivotal role in establishing the Pakistan Medical Journalists Association in 1983 *(PMJA, 2006; see also http://www.pmja.com/)*. It has, over the years, organized a large number of seminars and symposia on medical writing for young writers, trainees and resident staff, besides the junior faculty members. *PMJA* also organized hands on workshops on Peer Review (Jawaid, 2002). It helped the Pakistan Medical and Dental Council to set some standards for medical and dental journals published from Pakistan, thereby improving the standard of medical journalism in this part of the world (Jawaid and Jafary, 1998; Jawaid and Jafary, 2002). I had the rare distinction of serving as Founder President of *PMJA* for almost a decade, and now it is established on sound footings with a democratic set-up. Elections are now held on a regular basis. *PMJA* has published over a dozen books on different subjects. Detailed information is available on its website *(http://www.pmja.com).*

When I published my first book on Medical Writing in 1993 (Jawaid and Jafary, 1993), I invited Major Gen. Mohsin Pal, a former Director General of Health in the Federal Health Ministry, to be the chief guest at the formal launching ceremony of this book. After some reluctance, he agreed. I still remember the pearls of wisdom from his speech. His words still ring in my ears:

Knowledge flows to those who are capable of receiving it, respecting it, protecting it, preserving it and promoting it.

It conveys a lot to those who are blessed with the capabilities of understanding it. It has a message for everyone, from writers to editors.

## Three Important Functions Of An Editor

To me an editor has three important functions to perform:

- Firstly, to *Inform* the readers what is happening in and around in his area of interest;
- Secondly, to *Educate* them; and
- Thirdly, to *Guide* them.

The editor performs all these tasks with the help, assistance and cooperation of the editorial team, including reviewers and referees. As with all other professionals, it is particularly important that, in order to educate and guide appropriately, the editor continues with his own professional development.

## Agony And Ecstasy Of Being An Editor

If, on the one hand, an editor has to face innumerable problems and difficulties, being a successful editor, while keeping up professional ethics, also offers lot of joy and pleasure. After having established one's credibility, it is indeed heartening to note that one can influence the decisions of an influential class like doctors, people in the pharmaceutical trade and industry, as well as the health officials and planners in the health sector.

During my professional career in the field of medical journalism which started in 1966, I have had to face a lot of court cases from doctors, medical institutions, receive legal notices, threatening phone calls and nasty communications from those whose unethical practices were exposed, or whose vested interests were highlighted. Apart from suffering financial losses, my children had to pay the price for the ′sins′ and ′crimes′, which their father had committed, by exposing unethical practices of the examining bodies in the field of medical education. One must develop the courage to remain unruffled in such circumstances.

Being the editor of a journal provided me with opportunities to share my knowledge and experience at various national and international forums. My contributions have been appreciated and recognized by peers in this field. I have been able to help a large number of faculty members and others interested in writing, training many youngsters in the art of medical writing, and have so far authored thirteen books on different subjects. I am also proud of the role, which I, as a medical editor, played in highlighting the unethical medical practices of the medical profession and unethical marketing practices of the pharmaceutical trade and industry. This lead to the formation of a National Bioethics Committee by the Federal Health Ministry, Government of Pakistan, of which I am one of the sixteen members (Constitution of National Bioethics Committee, 2004). Here, again, when I started investigating few issues of unethical marketing practices by the Pharma industry, the Federal Health Ministry took notice and issued notices to a few companies.

An editor has to be courageous enough to take up controversial issues. He should not be afraid of any criticism of his decisions. Late Prof. Najib Khan was an eminent medical educationist who served as Principal of Nishtar Medical College in Multan, was founder of Liaquat Medical College Jamshoro Hyderabad in Sindh Province and retired as Prof. of Medicine from Dow Medical College in Karachi. He often used to say:

*Only those who are dead or do nothing have no critics*.

I feel satisfied, since I have always tried my best to uphold professional ethics.

Seizing the opportunities that came my way, I have been able to develop regional co-operation and collaboration. The fact that now our journal, *Pakistan Journal of Medical Sciences,* attracts manuscripts from over two-dozen countries from all the four continents is a testimony to the fact that we have been able to achieve successes in our journey, in our march forward, trying to still persevere to achieve the very best.

At the end of the day, it has been much more than satisfying to find out that there is no dearth of people in the medical profession, as well as the pharmaceutical industry, who wish to know nothing but the truth. Hence, unearthing the truth not only provided me professional satisfaction but also tremendous love and respect in society. All this is not a mean achievement.

## Conclusions

1. Local dose finding studies should be a must for registration before any new drug is marketed.
2. Rejection of a manuscript by a *Medline* indexed journal does not mean that it does not merit publication.
3. Pharma industry in general and multinationals in particular usually do not give importance to adverse drug reactions/events reported by physicians in the developing third world countries, a practice which needs to be changed.
4. One does not have to be a medical heavy weight, senior consultant or professor to note adverse drug reactions. Any caring conscious intelligent healthcare professionals can observe it.
5. There is a need for establishing National Adverse Drug Reactions centers in all the developing third world countries, which do exist in the West in most developed countries.
6. Financial viability of publishing good quality peer reviewed medical journals from developing third world countries is a serious problem which needs to be resolved by looking at some means of generating revenue, grants from various institutions etc.
7. Medicine and journalism are noble professions. Those infected with the materialistic virus should opt for some other profession, which will allow them lot of opportunities to make quick money.

## In Closing

For an editor, financial considerations should never come in his way while performing his duties. Even in today's materialistic world, it is my firm belief that one can earn a decent living while keeping up professional ethics. One has only to be a contented person and carefully watch the dividing line between *need* and *greed*. God Almighty has always been kind to me. I am myself a very contented person and God has blessed me with a family, which is all very contented. This provides me lot of strength to perform my duties with devotion. It is the support of my family and the understanding which they showed, coupled with the assistance and guidance of my friends, well-wishers in the medical profession, and also pharmaceutical manufacturers who believe and practice ethical marketing and continue to place advertisements in our publications (this being the major source of revenue) - all these have helped me achieve the present position.

## Questions That This Paper Raises

1. Pharmaceutical industry is the major source of revenue for medical journals through advertising. At times the journals are faced with a dilemma when the industry tries to influence the editorial policy as regards publication of clinical trials or adverse drug reactions. What course should medical journals pursue to ensure an independent policy while at the same time ensuring sustained continued publication?
2. Local dose finding studies are now compulsory in many countries before registration of a new drug. If any healthcare professional observes some unwanted side effects even after local dose finding studies have declared the drug to be safe, should it be discarded, or it is better to have another look at the safety of the drug and earlier dose finding studies?
3. Simultaneous submission of manuscripts to more than one periodical by authors is a serious problem faced by medical journals. Editors cannot act as policemen and they have to trust the authors when they give a written undertaking regarding exclusive submission. Even after giving a written undertaking, if an author indulges in unethical practice of simultaneous submission, what can the editors do to discourage this practice apart from black listing such authors and conveying this to their employers, institutions they are affiliated with, as well as concerned government agencies?
4. If a manuscript is rejected by an indexed journal, it does not mean this does not merit publication. If the authors submit this manuscript to another journal, should it be entertained and published after proper peer review? Secondly, is it essential for the authors to disclose that this manuscript was earlier submitted to another journal and the reasons why it was rejected? Thirdly, will such a disclosure affect the decision of the editors to accept or reject the manuscript?
5. Doctors in developing third world countries are usually afraid to report any adverse drug reactions because they fear losing the cozy relationship they enjoy with the pharma industry which offers them many benefits. This is an important reason why setting up ADR Monitoring Centers has not been a success. What measures should be taken to encourage and convince them that such adverse drug reactions are regularly reported to concerned ADR monitoring centers and agencies so as to save the patients unnecessary side effects which increase morbidity, and at times could prove fatal?

## About the Author


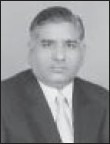


*Shaukat Ali Jawaid is one of the senior most medical journalists of Pakistan active in the field of medical journalism since 1966. Currently he is Chief Editor,* Pulse International, *a fortnightly medical newspaper, and Managing Editor, Pakistan Journal of Medical Sciences, a peer reviewed medical journal. He is former Founder President of Pakistan Medical Journalists Association, Member British Medical Journalists Association, and Vice President at large of Eastern Mediterranean Association of Medical Editors (EMAME). He is also General Secretary of Pakistan Aspirin Foundation and Member, National Bioethics Committee constituted by Government of Pakistan. He has authored thirteen books on topics like Medical Writing, Medical Ethics in Contemporary Era, Aspirin- the life saving miracle drug, Medical Men and Women of Pakistan etc. He is regularly invited as facilitator at workshops and seminars on Medical Writing and Medical Ethics. He has thirty scientific papers to his credit.*

## Footnotes

* Editor's note: The author (SAJ) states: “I do not have any original reference to the quotation from Dr. Hugh Clegg, former Editor of the British Medical Journal. I took it from the presentation of a European speaker at the International conference on urology and transplantation at Karachi, about eight years ago, who had shown this slide.”

* jahez means dowry (eds).
